# Properties of Maxentropic DNA Synthesis Codes †

**DOI:** 10.3390/e26121028

**Published:** 2024-11-27

**Authors:** Kees Schouhamer Immink, Jos H. Weber, Kui Cai

**Affiliations:** 1 Turing Machines Inc., Willemskade 15, 3016 DK Rotterdam, The Netherlands; 2 Department of Applied Mathematics, Delft University of Technology, 2628 CD Delft, The Netherlands; j.h.weber@tudelft.nl; 3 Science, Mathematics and Technology Cluster, Singapore University of Technology and Design (SUTD), 8 Somapah Rd, Singapore 487372, Singapore; cai_kui@sutd.edu.sg

**Keywords:** code design, DNA synthesis, low-weight code, maximum runlength constraint, nibble replacement (NR) code

## Abstract

Low-weight codes have been proposed for efficiently synthesizing deoxyribonucleic acid (DNA) for massive data storage, where a multiple of DNA strands are synthesized in parallel. We report on the redundancy and information rate of maxentropic low-weight codes for asymptotically large codeword length. We compare the performance of low-complexity nibble replacement (NR) codes, which are designed to minimize the synthesis time, with the performance of maxentropic low-weight codes. Finally, the asymptotic redundancy and information rate of codes with a runlength limitation are investigated.

## 1. Introduction

The pioneering experiments conducted by Church et al. [1] demonstrated the feasibility to store data in synthetic deoxyribonucleic acid (DNA), promising a huge data capacity, nil dissipation during storage, and very long-term stability. Natural DNA consists of four types of nucleotides: adenine (‘A’), cytosine (‘C’), guanine (‘G’), and thymine (‘T’). Codes are used for translating user data into sequences of digits in the quaternary alphabet {A, C, G, T} that are suitable for the synthesis of DNA strands. Prior art studies have focused on error-correcting codes for restoring various kinds of defects in DNA [2,3,4] or constrained codes that avoid the generation of vexatious DNA sequences that are prone to error; see, for example, Refs. [5,6,7,8].

The synthesis of DNA strands is a relative expensive part of the storage chain. In array-based synthesis, multiple DNA strands are synthesized in parallel [9] by adding in each cycle a single nucleotide to a subset of the DNA strands. Lenz et al. [10], Makarychev et al. [11], Elishco and Huleihel [12], Immink et al. [13], and Nguyen et al. [14] presented and analyzed coding techniques for efficiently synthesizing multiple parallel strands so that overall synthesis time can be shortened. Of specific interest in minimizing the synthesis time are sets (codes) of words of low weight, which are dealt with in the next subsection.

### 1.1. Low-Weight Codes

Although our main interest is in the quaternary DNA case, we will consider *q*-ary sequences for generality. For clerical convenience, we assume that the alphabet used is {1,…,q}, where q>1 is a positive integer. For the DNA case, we represent the quaternary alphabet {A, C, G, T} by {1,…,4}. Let $a=(a_{1},\ldots ,a_{n})$, $a_{i}\in \left\{1,\ldots ,q\right\}$, be a sequence of *n* symbols, called *word* of length *n*. The symbol sum

(1) $$w\left(a\right)=\sum_{i=1}^{n}a_{i}$$

is termed the *weight* of the word ***a***. Clearly, n≤w(a)≤qn. A *constant-weight code* of length *n*, denoted by $S_{n}\left(w\right)$, consists of all words of weight *w*, that is,

(2) $$S_{n}\left(w\right)=\left\{a\in {\left\{1,\ldots ,q\right\}}^{n}:w\left(a\right)=w\right\}.$$

The size of $S_{n}\left(w\right)$, denoted by $|S_{n}\left(w\right)|$, is found as the coefficient of $z^{w}$ of the *generating function* [15]

(3) $${\left(\sum_{i=1}^{q}z^{i}\right)}^{n}.$$

For synthesizing multiple words into physical sequences in parallel, we assume the sequences are generated by adding symbols in cycles. In each cycle in the synthesis process, one particular symbol from {1,…,q} is added to the sequences of the words waiting for that symbol. Throughout this paper, we assume the symbol adding in the subsequent cycles is in the order 1,2,…,q,1,2,…,q,1,2,…, which has been shown to be optimal; see [10,12]. In order to allow any word from ${\left\{1,\ldots ,q\right\}}^{n}$ to be synthesized, qn cycles are needed. By restricting the set of words used for representing data, the number of required synthesis cycles can be reduced, as explained next.

Let the *low-weight code* $\cup_{w=n}^{t}S_{n}\left(w\right)$ be the union of the sets of words of weight w≤t, where the integer *t*, n≤t≤qn denotes the maximum weight of the codewords. As explained in [10,13], low-weight codewords ***y*** can be bijectively mapped to words $x=(x_{1},\ldots ,x_{n})$, $x_{i}\in \left\{1,\ldots ,q\right\}$, by

(4) $$x_{i}=x_{i-1}+y_{i}\;\mathrm{mod}\;q,$$

with $x_{0}=q$, such that the words ***x*** have a synthesis time of at most *t* cycles. Let the low-weight code be denoted as $Y_{n}\left(\gamma \right)$, where γ=t/n, and the associated set of words ***x*** as $X_{n}\left(\gamma \right)$. From the synthesis perspective, we are interested in properties of the codes $X_{n}\left(\gamma \right)$, but because of the bijective mapping we can also study the low-weight codes $Y_{n}\left(\gamma \right)$.

### 1.2. Redundancy and Information Rate

The *redundancy* (in bits per symbol) of a low-weight code $Y_{n}\left(\gamma \right)$ is defined by

(5) $$\rho_{n}\left(\gamma \right)=\log_{2}\left(q\right)-R_{n}\left(\gamma \right),$$

where

(6) $$R_{n}\left(\gamma \right)=\frac{1}{n}\log_{2}\left|Y_{n}\left(\gamma \right)\right|.$$

Lenz et al. [10] also introduced the *information rate* (in bits per cycle) of a low-weight code $Y_{n}\left(\gamma \right)$ as

(7) $$W_{n}\left(\gamma \right)=\frac{1}{n\gamma }\log_{2}\left|Y_{n}\left(w\right)\right|=\frac{1}{\gamma }R_{n}\left(\gamma \right).$$

Of course, $\rho_{n}\left(\gamma \right)$ and $W_{n}\left(\gamma \right)$ are also the redundancy and information rate, respectively, of $X_{n}\left(\gamma \right)$. Note that $W_{n}\left(\gamma \right)$ is a measure for the synthesis efficiency of the codewords of $X_{n}\left(\gamma \right)$.

**Figure 1:**
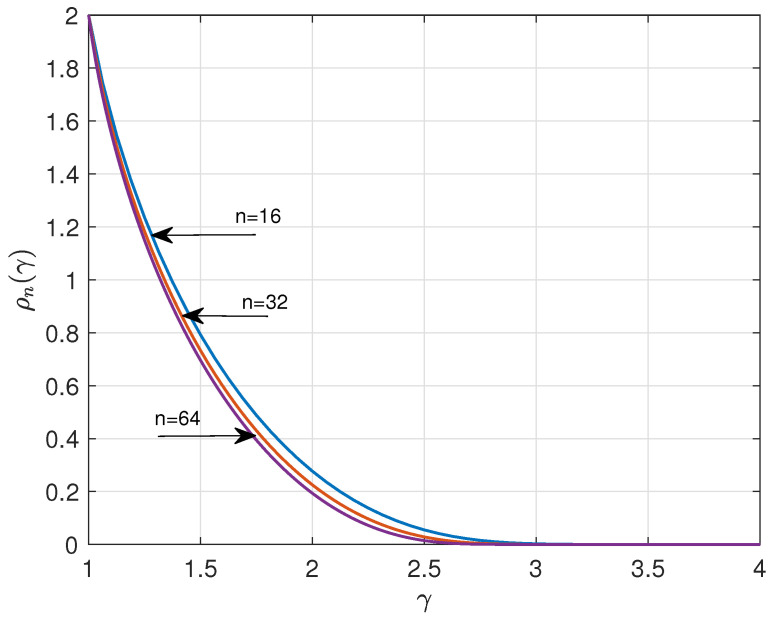
Redundancy $\rho_{n}\left(\gamma \right)$ versus γ for n=16,32,64, and q=4.

**Figure 2:**
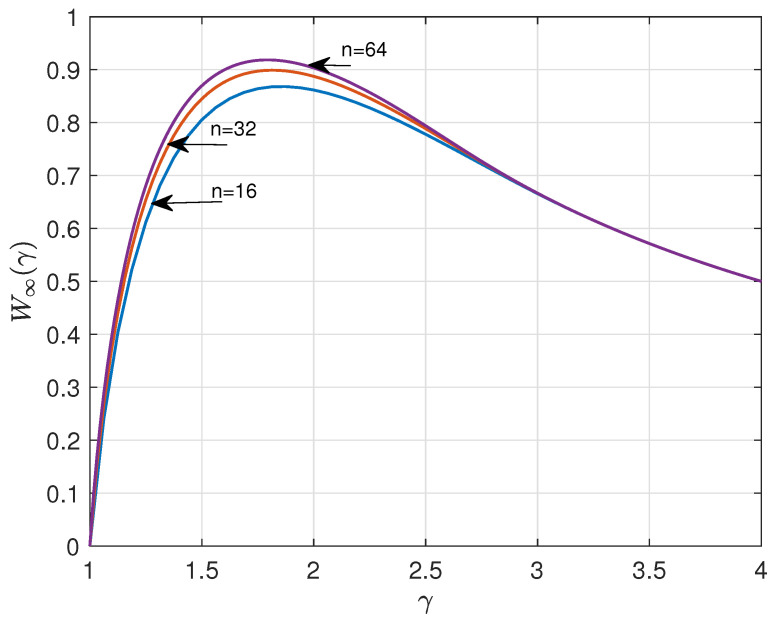
Information rate $W_{n}\left(\gamma \right)$ versus γ for n=16,32,64, and q=4.

Using (3), we can straightforwardly compute $\rho_{n}\left(\gamma \right)$ and $W_{n}\left(\gamma \right)$ versus γ. Figure 1 and Figure 2 show the results for n=16, 32, 64, and q=4. The curves suggest that $\rho_{n}\left(\gamma \right)$ and $W_{n}\left(\gamma \right)$ have a lower bound and upper bound, respectively, for asymptotic large *n*. A major goal of this paper is to determine these bounds.

### 1.3. Contributions and Overview of the Paper

Besides introducing the framework as just described, Lenz et al. [10] also conducted a brief performance analysis of DNA synthesis codes, mainly based on tools from the theory of cost-constrained channels. Constructions of efficient DNA synthesis codes were further explored in [13]. Here, in this paper, Section 2 deals with an extensive asymptotic analysis, with a focus on the trade-off between redundancy and information rate. The results are derived using Jaynes’ maximum entropy principle. In Section 3, we compare the obtained theoretical optima with the performance of practical nibble replacement codes. Finally, we extend the analysis to codes with a runlength constraint in Section 4 and conclude the paper in Section 5.

## 2. Asymptotic Analysis of Low-Weight Codes

In order to evaluate the sizes of large low-weight codes, we use the following approach. Let $C_{n}\left(w\right)$ be the set of compositions $c=(n_{1},\ldots ,n_{q})$ of *n*, where $n_{i}$ are nonnegative integers such that $\sum_{i=1}^{q}n_{i}=n$ subject to the constraint $\sum_{i=1}^{q}in_{i}=w$. The number of *q*-ary words of length *n* with $n_{i}$ symbols equal to *i*, 1≤i≤q, denoted by $N_{c}$, equals

(8) $$N_{c}=\frac{n!}{n_{1}!\ldots n_{q}!}.$$

The constant-weight code size, $|S_{n}\left(w\right)|$, is found by summing $N_{c}$ for all possible compositions $c\in C_{n}\left(w\right)$ so that

(9) $$|S_{n}\left(w\right)|=\sum_{c\in C_{n}\left(w\right)}N_{c}.$$

### 2.1. Asymptotic Analysis of $R_{n}\left(\gamma \right)$

We are specifically interested in $R_{n}\left(\gamma \right)$ for asymptotically large *n*. So, let n→∞ and $n_{i}\to \infty$ for all *i*, while keeping $p_{i}=n_{i}/n$, 1≤i≤q, the distribution of the symbol values, fixed. It then follows (see Wallis argument in Section 11.4 of [16]), using Stirling’s approximation, that

(10) $$\frac{1}{n}\log_{2}N_{c}\to H_{c},$$

and thus

(11) $$\frac{1}{n}\log_{2}\left|S_{n}\left(w\right)\right|\to \max_{c\in C_{n}\left(w\right)}H_{c},$$

where

(12) $$H_{c}=-\sum_{i=1}^{q}p_{i}\log_{2}p_{i}.$$

In a similar vein, we find

(13) $$R_{n}\left(\gamma \right)\to \max_{n\leq w\leq \gamma n}\frac{1}{n}\log_{2}\left|S_{n}\left(w\right)\right|\to \max_{n\leq w\leq \gamma n}\max_{c\in C_{n}\left(w\right)}H_{c}.$$

Since $\frac{1}{n}\log_{2}\left|S_{n}\left(w\right)\right|$ is monotonically increasing with *w*, n≤w≤γn, if 1≤γ≤(q+1)/2, with a maximum $\log_{2}\left(q\right)$ at w=n(q+1)/2, we infer

(14) $$R_{n}\left(\gamma \right)\to \left\{\begin{array}{cc} \max_{c\in C_{n}\left(\gamma n\right)}H_{c}, & 1\leq \gamma <(q+1)/2,\\ \log_{2}\left(q\right), & (q+1)/2\leq \gamma \leq q. \end{array}\right.$$

The problem of determining $R_{\infty }\left(\gamma \right)=\lim_{n\to \infty }R_{n}\left(\gamma \right)$, and thus the asymptotic redundancy $\rho_{\infty }\left(\gamma \right)=\log_{2}\left(q\right)-R_{\infty }\left(\gamma \right)$ and the asymptotic information rate $W_{\infty }\left(\gamma \right)=\frac{1}{\gamma }R_{\infty }\left(\gamma \right)$, is now a matter of finding, for asymptotically large *n*, a c in $C_{n}\left(\gamma n\right)$ that maximizes $H_{c}$. The composition $c=(n_{1},\ldots ,n_{q})$ in $C_{n}\left(\gamma n\right)$ is characterized by

(15) $$\sum_{i=1}^{q}n_{i}=n\;\mathrm{and}\;\sum_{i=1}^{q}in_{i}=\gamma n,$$

which can be conveniently rewritten as

(16) $$\sum_{i=1}^{q}p_{i}=1\;\mathrm{and}\;\sum_{i=1}^{q}ip_{i}=\gamma .$$

In the next subsection, we maximize $H_{c}$ by a judicious choice of the distribution of the symbol values, $p_{i}$, under these conditions.

### 2.2. Principle of Maximum Entropy

We change the above setting of finite-length codewords and now assume a stationary information source that transmits symbols of (integer) magnitude *i*, i∈{1,…,q}, with probability distribution p=$\left(p_{1},\ldots ,p_{q}\right)$, where $p_{i}\in R$ and $\sum p_{i}=1$. The information content per symbol sent, or *entropy*, denoted by *H*, defined by Shannon [17], is

(17) $$H=-\sum_{i=1}^{q}p_{i}\log_{2}p_{i}.$$

Although the variable $H_{c}$ in (12) and Shannon’s entropy *H* share the same expression in ***p***, the background of the expressions is different [16]. Note that in (12), the $p_{i}$’s are rational numbers, while in (17) the $p_{i}$’s are assumed to be real numbers.

We are interested in maximizing the entropy *H*. Define

(18) $$\hat{H}\left(\gamma \right)=\max_{p_{1},\ldots ,p_{q}}H,$$

1≤γ≤q, where the maximization over the $p_{i}$ is under the conditions (16). Jaynes [18] concluded that the entropy, *H*, is maximized subject to these constraints by the maxentropic probability distribution

(19) $${\hat{p}}_{i}=2^{\alpha -\beta i},\;\;1\leq i\leq q,$$

where the parameters α and β, α,β∈R, satisfy the conditions

(20) $$\alpha =-\log_{2}\sum_{i=1}^{q}2^{-\beta i}$$

and

(21) $$\sum_{i=1}^{q}i2^{\alpha -\beta i}=\gamma .$$

After substituting (19) to (21) into (17), we find

(22) $$\hat{H}\left(\gamma \right)=\beta \gamma -\alpha .$$

For the case q=2, we may easily find that $p_{1}+p_{2}=1$ and $p_{1}+2p_{2}=\gamma$, so that $p_{1}=2-\gamma$, $p_{2}=\gamma -1$, and

(23) $$\hat{H}\left(\gamma \right)=-\left(2-\gamma \right)\log_{2}\left(2-\gamma \right)-\left(\gamma -1\right)\log_{2}\left(\gamma -1\right),$$

1≤γ≤2. For q>2, no simple closed-form expression could be found, and we use numerical methods for solving (20) and (21).

### 2.3. Asymptotic Redundancy and Information Rate

From (14), we obtain

(24) $$R_{\infty }\left(\gamma \right)=\left\{\begin{array}{ll} \hat{H}\left(\gamma \right), & 1\leq \gamma <(q+1)/2,\\ \log_{2}\left(q\right), & (q+1)/2\leq \gamma \leq q. \end{array}\right.$$

As a result, the asymptotic redundancy is

(25) $$\rho_{\infty }\left(\gamma \right)=\left\{\begin{array}{cc} \log_{2}\left(q\right)-\hat{H}\left(\gamma \right), & 1\leq \gamma <(q+1)/2,\\ 0, & (q+1)/2\leq \gamma \leq q. \end{array}\right.$$

**Figure 3:**
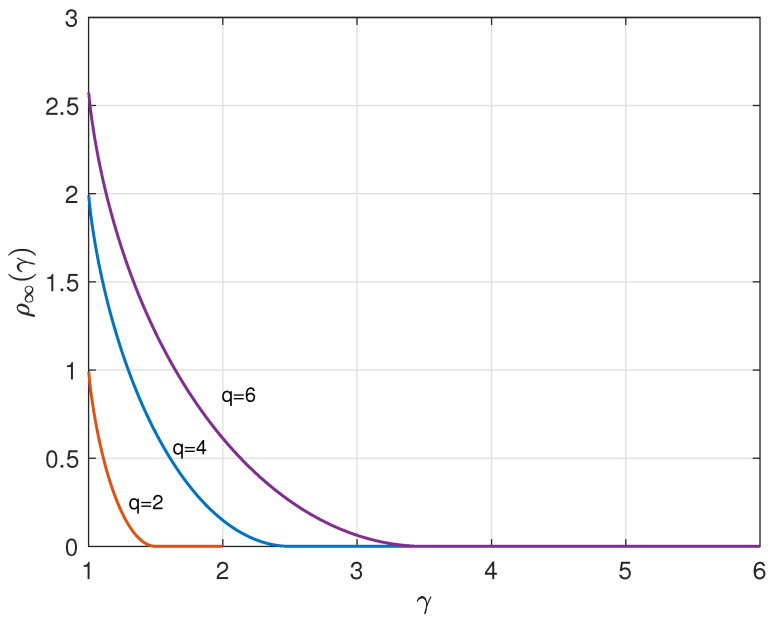
Redundancy $\rho_{\infty }\left(\gamma \right)$ versus γ, q=2,4, and 6.

Figure 3 depicts, for q=2,4, and 6, the relationship between the asymptotic redundancy $\rho_{\infty }\left(\gamma \right)$ and γ.

The asymptotic information rate, $W_{\infty }\left(\gamma \right)$, equals

(26) $$W_{\infty }\left(\gamma \right)=\left\{\begin{array}{cc} \hat{H}\left(\gamma \right)/\gamma , & 1\leq \gamma <(q+1)/2,\\ \log_{2}\left(q\right)/\gamma , & (q+1)/2\leq \gamma \leq q. \end{array}\right.$$

**Figure 4:**
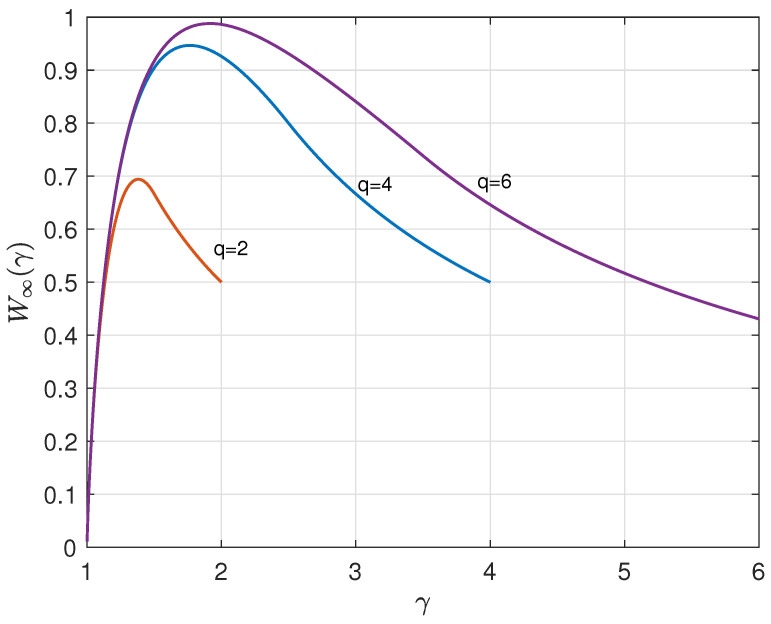
Information rate $W_{\infty }\left(\gamma \right)$ versus γ, q=2,4, and 6.

Figure 4 shows $W_{\infty }\left(\gamma \right)$ versus γ for q=2,4, and 6.

The maximum asymptotic information rate, denoted by

$${\hat{W}}_{\infty }=\max_{\gamma }W_{\infty }\left(\gamma \right),$$

can be found after an analysis of (22). We write (22), using (20) and (21), as a function of β and conclude that the largest (real) root of

(27) $$\sum_{i=1}^{q}2^{-i\beta }=1,$$

denoted by $\hat{\beta }$, maximizes $W_{\infty }\left(\gamma \right)$. We obtain, see (20), α=0 and hence, see (22), we infer that

(28) $${\hat{H}}_{\infty }=\hat{H}\left(\hat{\gamma }\right)=\hat{\beta }\hat{\gamma },$$

where

(29) $$\hat{\gamma }=\sum_{i=1}^{q}i2^{-\hat{\beta }i},$$

and

(30) $${\hat{W}}_{\infty }=\hat{\beta }.$$

Note that Equation (27) is equivalent to the characteristic equation $\sum_{i=1}^{q}z^{-i}=1$ of a binary source under the constraint that the maximum runlength is *q* [19]. The capacity of binary sequences with a maximum runlength constraint of *q* equals $\log_{2}\left(\hat{z}\right)$, where $\hat{z}$ is largest (real) root of the characteristic equation [17]. Hence, the maximum asymptotic information rate ${\hat{W}}_{\infty }=\hat{\beta }$ of *q*-ary low-weight codes is equal to this capacity. Numerical values of the latter have been listed for selected values of *q* in [19]. Since the capacity approaches unity for increasing values of *q*, the same holds for the information rate ${\hat{W}}_{\infty }$, which is achieved for $\hat{\gamma }\to 2$. In other words, for large values of *n* and *q*, the maximum information rate is achieved by setting the maximum weight of the low-weight code equal to (roughly) 2n. The corresponding redundancy is $\log_{2}\left(q\right)-\hat{\gamma }\hat{\beta }\to \log_{2}\left(q\right)-2$. For any *q*, the asymptotic redundancy can be lowered from $\log_{2}\left(q\right)-\hat{\gamma }\hat{\beta }$ to zero by increasing γ from $\hat{\gamma }$ to (q+1)/2, which implies that the asymptotic information rate decreases from $\hat{\beta }$ to $2\log_{2}\left(q\right)/\left(q+1\right)$. This trade-off between redundancy and information rate is further explored for the case q=4 in the next subsection.

### 2.4. Case Study for q=4

**Figure 5:**
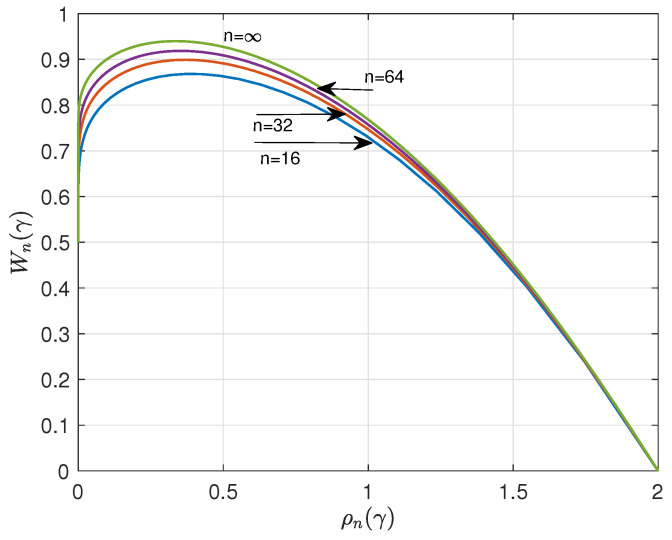
Parametric relationship between maxentropic information rate $W_{n}\left(\gamma \right)$ versus redundancy $\rho_{n}\left(\gamma \right)$, q=4. As a comparison, we plotted $W_{n}\left(\gamma \right)$ versus $\rho_{n}\left(\gamma \right)$ for n=16,32,64.

In this subsection, we consider the case q=4, which is of particular interest since it is the alphabet size for DNA synthesis codes. For q=4, we find using numerical methods that ${\hat{W}}_{\infty }=\hat{\beta }=0.947$, $\hat{\gamma }=1.766$, and ${\hat{H}}_{\infty }=1.672$. The probability distribution at maximum entropy is $\hat{p}=$ (0.519, 0.269, 0.140, 0.072). Figure 5 shows the parametric representation of $W_{\infty }\left(\gamma \right)$ versus $\rho_{\infty }\left(\gamma \right)$ with γ as a parameter for the case q=4. The curve is a typical price/performance curve, where we may observe that a higher $W_{\infty }\left(\gamma \right)$ comes with a higher penalty in redundancy $\rho_{\infty }\left(\gamma \right)$.

**Figure 6:**
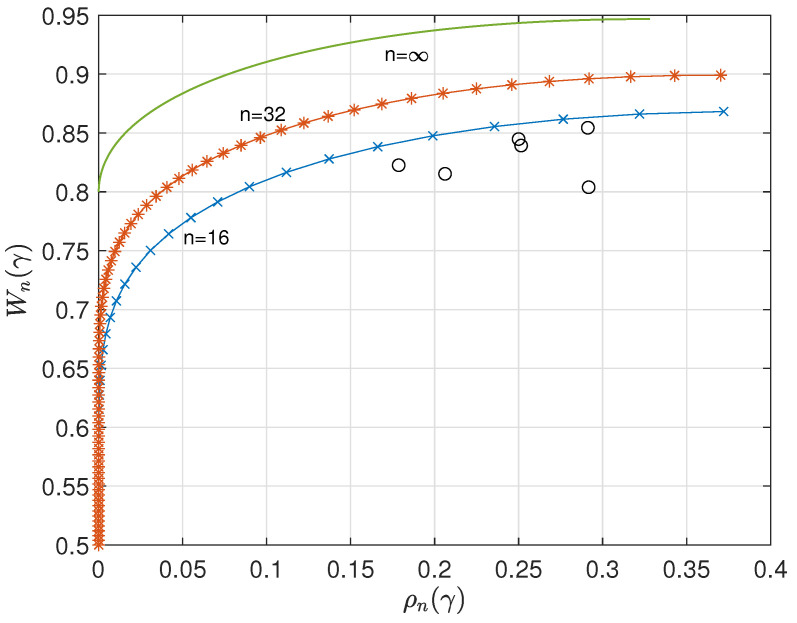
Parametric relationship between (maxentropic) information rate $W_{n}\left(\gamma \right)$ versus redundancy $\rho_{n}\left(\gamma \right)$, n=16,32,∞, q=4, in the range of practical interest. The black circles refer to the NR codes compiled in Table 1.

It is the difficult task of a system designer to trade the costs and benefits of the conflicting parameters. Note that in the range γ≥5/2 we have $\rho_{\infty }\left(\gamma \right)=0$, a zero-redundant system, while in the range $\gamma <\hat{\gamma }$ we may achieve the same information rate $W_{\infty }\left(\gamma \right)$ with a smaller redundancy. For example, we may notice that we can achieve $W_{\infty }\left(\gamma \right)=0.8$ for zero redundancy cost or for roughly 0.9. In practice, we prefer the smaller redundancy alternative so that in this range of practical interest, we have $4/5\leq W_{\infty }\left(\gamma \right)\leq {\hat{W}}_{\infty }=0.947$ and $0\leq \rho_{\infty }\left(\gamma \right)\leq 2-{\hat{H}}_{\infty }=0.328$. Figure 6 displays $W_{\infty }\left(\gamma \right)$ versus $\rho_{\infty }\left(\gamma \right)$ in the range of practical interest $\hat{\gamma }\leq \gamma <5/2$.

## 3. Comparison with Implemented Codes

In this section, we compare the performance of implemented codes with that of maxentropic low-weight codes. In [13], various code implementations have been assessed. Here, we focus on the *nibble replacement (NR) algorithm* [13,20], which is an efficient method for encoding/decoding with small complexity and redundancy.

In the NR format, an *n*-symbol strand is divided into *L* subwords of length *m*, so that n=Lm. Let $t_{m}$ be the maximum allowed cycle count of an *m*-symbol *q*-ary word, then the maximum cycle count of the *n*-symbol *q*-ary word is $t=Lt_{m}$. Let

(31) $$M=\sum_{w=m}^{t_{m}}\left|S_{m}\left(w\right)\right|$$

denote the number of low-weight *m*-symbol codewords. Define $m_{h}=\left\lceil \log_{2}M\right\rceil$ and

(32) $$L=\left\lfloor \frac{2^{m_{h}-1}}{2^{m_{h}}-M}\right\rfloor .$$

The NR algorithm translates $Lm_{h}-1$ source bits into *L* $m_{h}$-bit words. Each $m_{h}$-bit word is translated, using a look-up table, into a *q*-ary *m*-symbol word that satisfies the $t_{m}$-cycle count constraint. The NR encoding method requires data storage of *L* $m_{h}$-bit words, the execution of the encoding algorithm [13], and a look-up table for translating an $m_{h}$-bit wide word into a word of *m* *q*-ary symbols so that very large, *n*-symbol wide, look-up tables are avoided. The overall redundancy per symbol, ρ(t), and information rate, W(t), of the *n*-symbol word are

(33) $$\rho \left(t\right)=\frac{L(\log_{2}\left(q\right)m-m_{h})+1}{n}$$

and

(34) $$W\left(t\right)=\frac{Lm_{h}-1}{t}.$$

Table 1 shows numerical results selected from Table I in [13].

The scattered points (black circles) in Figure 6 are found by plotting the redundancy, ρ(t), and information rate, W(t), of the NR codes shown in Table 1.

## 4. Runlength Limitation

It is known that homopolymer runs, i.e., adjacent repetitions of the same nucleotide, make DNA-based data storage more error prone [12]. Therefore, it could be advantageous to use strands in which long runs are avoided. Of course, this comes at the expense of an increased redundancy. In this section, we perform an asymptotic analysis of codes aiming at (i) small redundancy, (ii) high information rate, and (iii) small maximum runlength. These are conflicting goals resulting into trade-off considerations. Again, we start by investigating *q*-ary codes and then focus on the q=4 case.

We say that a code is *r*-RLL (runlength limited) if within any codeword any run of identical symbols is of length at most *r*, where 1≤r≤n. When r=n, there is actually no constraint with respect to the runlength. Here, we focus on the other extreme, r=1; i.e., we consider codewords in which any two adjacent symbols are different. We investigate the asymptotic redundancy and information rate of *q*-ary 1-RLL codes. The same notation as before is used, where we indicate with a tilde that the r=1 constraint is in place.

Let ${\tilde{X}}_{n}\left(\gamma \right)$ denote the *q*-ary code consisting of all 1-RLL sequences that can be synthesized in at most t=γn cycles. The codewords $\tilde{y}=\left({\tilde{y}}_{1},\ldots ,{\tilde{y}}_{n}\right)$ of the associated low-weight code ${\tilde{Y}}_{n}\left(\gamma \right)$ are obtained from the codewords $\tilde{x}=\left({\tilde{x}}_{1},\ldots ,{\tilde{x}}_{n}\right)$ of ${\tilde{X}}_{n}\left(\gamma \right)$ by the bijective mapping

(35) $${\tilde{y}}_{i}={\tilde{x}}_{i}-{\tilde{x}}_{i-1}\;\mathrm{mod}\;q$$

with ${\tilde{x}}_{0}=q$. Note that due to the 1-RLL property of $\tilde{x}$, it holds that ${\tilde{x}}_{i}\neq {\tilde{x}}_{i-1}$ and thus ${\tilde{y}}_{i}\neq q$ for all 2≤i≤n. Hence, ${\tilde{Y}}_{n}\left(\gamma \right)=\cup_{w=n}^{\gamma n}{\tilde{S}}_{n}\left(w\right)$, where

(36) $${\tilde{S}}_{n}\left(w\right)=\left\{\tilde{y}\in {\left\{1,\ldots ,q\right\}}^{n}:w\left(\tilde{y}\right)=w\wedge {\tilde{y}}_{i}\neq q\forall i\geq 2\right\}$$

and the range for γ is in this case 1≤γ≤q−1+1/n, since the maximum number of cycles is (q−1)n+1 rather than qn due to the runlength constraint.

Similarly to what we did before, we next evaluate

(37) $${\tilde{R}}_{n}\left(\gamma \right)=\frac{1}{n}\log_{2}\left|{\tilde{Y}}_{n}\left(\gamma \right)\right|.$$

Since the symbol distribution $\left({\tilde{p}}_{1},\ldots ,{\tilde{p}}_{q}\right)$ satisfies, for any codeword in the low-weight code,

(38) $${\tilde{p}}_{q}\leq \frac{1}{n}\to 0$$

as n→∞, we can conclude that the value of ${\tilde{R}}_{\infty }\left(\gamma \right)$ in the *q*-ary case is equal to the value of $R_{\infty }\left(\gamma \right)$ in the (q−1)-ary case. Hence, it easily follows that

- The asymptotic redundancy ${\tilde{\rho }}_{\infty }\left(\gamma \right)$ in the *q*-ary 1-RLL case equals $\log_{2}\left(q/\left(q-1\right)\right)$ plus the asymptotic redundancy $\rho_{\infty }\left(\gamma \right)$ in the (q−1)-ary case without runlength restriction;
- The asymptotic information rate ${\tilde{W}}_{\infty }\left(\gamma \right)$ in the *q*-ary 1-RLL case equals the asymptotic information rate $W_{\infty }\left(\gamma \right)$ in the (q−1)-ary case without runlength restriction.

**Figure 7:**
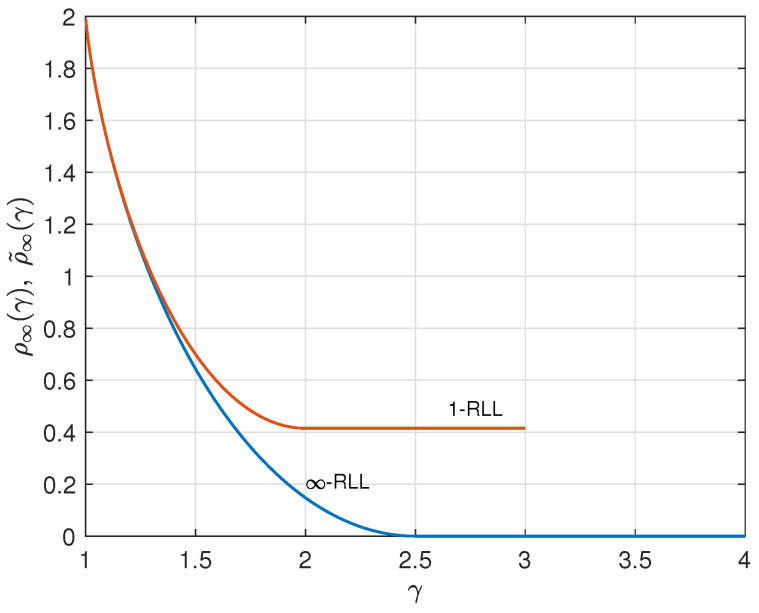
Asymptotic redundancies $\rho_{\infty }\left(\gamma \right)$ (*∞*-RLL) and ${\tilde{\rho }}_{\infty }\left(\gamma \right)$ (1-RLL) versus γ for the case q=4.

**Figure 8:**
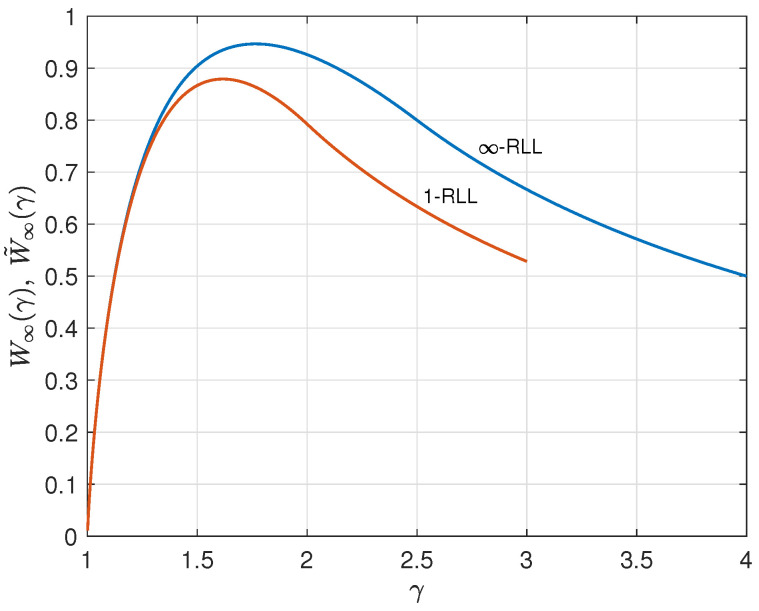
Asymptotic information rate $W_{\infty }\left(\gamma \right)$ (*∞*-RLL) and ${\tilde{W}}_{\infty }\left(\gamma \right)$ (1-RLL) versus γ for the case q=4.

**Figure 9:**
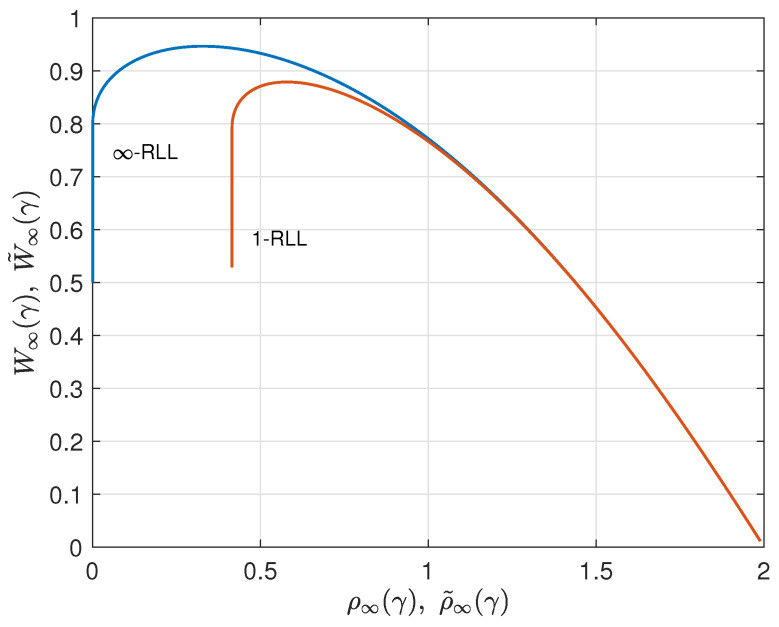
Parametric relationship between the asymptotic information rate and asymptotic redundancy for the *∞*-RLL and 1-RLL cases, q=4.

As an illustration, we consider the case q=4. By applying the results from (25) and (26) for q=3, we find ${\tilde{\rho }}_{\infty }\left(\gamma \right)$ and ${\tilde{W}}_{\infty }\left(\gamma \right)$ for q=4. These 1-RLL results are compared to the corresponding results without runlength limitation from Section 2 in Figure 7, Figure 8 and Figure 9. Results for *r*-RLL codes, 1<r<∞, will be in between the lower and the upper curves in these figures. Various trade-off possibilities can be considered. Note that, for small values of γ, imposing the runlength limitation comes at hardly any price, but that for larger values of γ we considerably pay in terms of redundancy and information rate. Fixing the asymptotic redundancy at, e.g., 0.5, it follows from Figure 9 that the asymptotic information rate drops from about 0.93 (*∞*-RLL, i.e., no runlength limitation) to about 0.87 (1-RLL).

## 5. Conclusions

We have analyzed coding techniques for efficiently synthesizing multiple parallel DNA strands. We have computed the maxentropic redundancy and information rate of low-weight codes, $\rho_{n}\left(\gamma \right)$ and $W_{n}\left(\gamma \right)$, for asymptotically large codeword length *n* using Jaynes’ maximum entropy principle. We have compared the performance of low-complexity NR codes, which are designed to minimize the synthesis time, with the performance of maxentropic low-weight codes. Finally, the performance of codes with a runlength limitation has been evaluated. All the presented results allow for making trade-offs between synthesis time and redundancy for long codes.

## Figures and Tables

**Table 1:** Results of the NR coding method for selected values of subword length *m* and maximum subword cycle count, $t_{m}$, q=4.

*m*	$t_{m}$	$m_{h}$	*L*	ρ(t)	W(t)
8	17	14	3	0.2917	0.8039
10	22	18	16	0.2062	0.8153
12	25	21	56	0.2515	0.8393
14	31	26	2	0.1786	0.8226
14	29	25	2	0.2500	0.8448
14	28	24	13	0.2912	0.8544

## Data Availability

The original contributions presented in the study are included in the article, further inquiries can be directed to the corresponding author.
